# Ovarian transposition before radiotherapy in cervical cancer patients: functional outcome and the adequate dose constraint

**DOI:** 10.1186/s13014-019-1312-2

**Published:** 2019-06-10

**Authors:** Lina Yin, Saiquan Lu, Jun Zhu, Weiling Zhang, Guihao Ke

**Affiliations:** 1 Department of Gynecologic Oncology, Fudan University Shanghai Cancer Center, Shanghai, China; 2 Department of Oncology, Shanghai Medical College, Fudan University, Shanghai, China; 3 Department of Radiation Oncology, Fudan University Shanghai Cancer Center, Shanghai, China; 4 Department of Gynecologic Oncology, Nantong Tumor Hospital, Nantong University, Nantong, China

**Keywords:** Cervical cancer, Ovarian transposition, Radiotherapy, Ovarian function

## Abstract

**Background:**

The data regarding a transposed ovary in intensity-modulated radiotherapy (IMRT) are not sufficient. Here we aim to investigate the adequate dose constraint of ovarian transposition before radiotherapy in cervical cancer patients.

**Methods:**

This was a retrospective analysis of 118 patients with cervical cancer who received a radical hysterectomy and ovarian transposition before pelvic irradiation from April 2012 to July 2017. A total of 105 patients underwent IMRT with a limited radiation dose to the ovaries; 48 of these patients received unilateral ovary limitation, while 57 received bilateral ovary limitations. Patient follow up regarding sex hormone levels (estrogen [E2], follicle stimulating hormone [FSH]) and menopausal symptoms was completed one year after their radiation therapy.

**Results:**

A total of 41 out of 105 patients (39.0%) who underwent IMRT with a limited radiation dose to the ovaries preserved their normal ovarian function. The cutoff dose of comparatively lower side ovarian maximum dose was 9.985Gy and the cutoff of mean dose was 5.32Gy. The optimal dose–volume constrains to ovaries was V5.5 < 29.65%. Age ≤ 38 (*P* = 0.001) was an independent predictors of ovarian function, while limited ovarian side numbers were excluded.

**Conclusion:**

Using IMRT, preservation of ovarian function was possible when the limited dose was as low as possible to the ovaries regardless of bilateral or unilateral limitation to the ovaries. The ovarian maximum dose of less than 9.985Gy, the mean dose less than 5.32Gy and V5.5 < 29.65% could be better at preventing ovarian dysfunction. Patients younger than 38 years old were more likely to keep normal ovarian function while limited ovarian side numbers did not appear to exert an obvious effect.

## Background

The number of cases of cervical cancer in young patients is growing. There were significant, increasing trends in cervical cancer mortality rates among young Japanese women below the age of 50 from 1975 to 2012 [1]. Cervical cancer incidence among women 20–24 years old increased significantly for New Zealand women(1985–2013) [2]. In Korea, the incidence and mortality rates of cervical cancer among young women (< 30 years old) increased from 1993 to 2012 [3]. According to statistics from 2010, in China, nearly 15.7% of cervical cancer cases occurred in women younger than 40 [4]. For patients diagnosed with locally advanced cervical cancer, the standard treatment recommendation is concurrent pelvic radiation therapy and chemotherapy [5]. In addition, postoperative adjuvant radiotherapy for patients with high-risk pathological factors is recommended by the National Comprehensive Cancer Network (NCCN) Guidelines for Cervical Cancer Version 2017. Unfortunately, radiation may cause ovarian function failure. Reduction of radiation exposure is used to avoid radiation-induced ovarian failure that can occur at cumulative doses of 600 to 2000 cGy [6]. Decreased ovarian function may not only lead to menopausal symptoms but may also cause osteoporosis, cardiovascular disease, and genitourinary atrophy. Because of these adverse events, some premenopausal patients with cervical cancer are recommended to undergo ovarian transposition [7–9].

Ovarian transposition may decrease the likelihood of ovarian dysfunction after treatment in young patients scheduled to undergo chemotherapy and pelvic irradiation. Typically, the ovaries are fixed in the paracolic gutters at the level of pelvic brim with a sufficient angle to maintain adequate blood supply [10]. In the era of 2D opposite-field radiotherapy, the standard recommended ovarian position during radiotherapy is 4 cm outside of the radiation field or more than 1.5 cm above the iliac crest [11].

The ovaries are extremely sensitive to radiation. The estimated scatter dose to the ovaries was directly related to the preservation of ovarian function. If the dose to the ovaries is limited to 300 cGy or less, only 1 out of 9 patients (11%) underwent menopause; however, 3 out of 5 patients (60%) became menopausal if the ovarian dose was more than 300 cGy [12]. A radiation dose of 250–300 cGy could inhibit ovarian function, and 500–1500 cGy of radiation induced temporary sex hormone disorder and infertility. Furthermore, if the radiation dose went up to 2000–3000 cGy, irreversible damage to the ovaries occurred [13]. These data are estimated based on data from the era of 2D opposite-field radiotherapy. The data regarding a transposed ovary in intensity-modulated radiotherapy (IMRT) are not sufficient. The purpose of this study was to analyze transposed ovarian dose limitation in IMRT.

## Methods

This was a retrospective analysis of 118 patients with cervical cancer who received radical hysterectomy and ovarian transposition before pelvic irradiation from April 2012 to December 2017 at the Fudan University Shanghai Cancer Center. The age of the patients ranged from 24 to 49. According to 2009 FIGO criteria, the clinical staging of tumors was Ib1~IIb. Postoperative adjuvant IMRT with dose of planning target volume (PTV) 4500–5000Gy/25–28f/5w was recommended for all 118 patients with high-risk pathological factors. In all cases, adjuvant, concurrent chemotherapy included 40 mg/m^2^ of cisplatin administered weekly for 4–6 weeks. A total of 13 patients received IMRT with no limitation on radiation dose to the ovaries. A total of 105 patients underwent IMRT with a limited radiation dose (as low as possible) to the ovaries, and 48 of these patients received unilateral ovary limitation while 57 received bilateral ovaries limitation. During the follow up, ovarian function was evaluated by measuring levels of FSH and E2 serum one year after the completion of pelvic irradiation. Ovarian function was considered to be preserved when the last follow up level, without hormone replacement therapy, of FSH was< 40 mIU/mL and E2 > 50 pg/mL, and patients displayed no menopausal symptoms. The influence of ovarian maximum dose, ovarian mean dose and age upon transposed ovarian function was also evaluated.

All calculations were done using the SPSS 23.0 statistical software package (SPSS Inc. Chicago, IL). Receiver operator characteristic (ROC) analysis and 95% confidence interval (CI) were used to analyze function of transposed ovaries and to determine the optimum cutoff point. Youden index = sensitivity+specificity-1. Youden index were calculated based on ROC table and the dose corresponding to the largest Youden index was defined as the cutoff point of dose constraints.

A two-sided *P* value of < 0.05 was considered a significant calculation of the optimal limited radiation dose that would preserve ovarian function. Multivariate analysis was used to analyze the relationship between covariates and normal ovarian function after lateral ovarian transposition. A two-sided P value of < 0.05 was considered significant.

## Results

Table 1 shows the characteristics of 118 patients. According to the 2009 FIGO criteria, clinical staging of the tumors identified 51 cases of Ib1, 18 cases of Ib2, 20 cases of IIa1, 15 cases of IIa2 and 14 cases of IIb. The median age of patients was 38 years, and the average was 37.93 years. Histological examination identified 104 cases of squamous cell carcinoma, 7 cases of adenocarcinoma, and 7 cases of other cancers.

**Table 1:** Patient characteristics

Patient characteristics	*N* = 118
Age (Range)	38 (24–49)
FIGO staging	
Ib1	51
Ib2	18
IIa1	20
IIa2	15
IIb	14
Histological types	
Squamous cell carcinoma	104
Adenocarcinoma	7
Others	7

Table 2 shows the comparison of ovarian function in patients undergoing different limitations of radiation. Ovarian transposition was performed on one ovary in 11 cases and on both ovaries in 107 cases. Ovarian function was absent in 13 patients who received IMRT with no limitation on radiation dose to the ovaries. A total of 41 cases out of 105 patients (39.0%) who underwent IMRT with a limited radiation dose (as low as possible) to the ovaries had preserved, normal ovarian function. The percentage of patients with normal ovarian function was 33.3 and 43.9% in unilateral and bilateral ovaries limitation (*P* = 0.318), respectively. In unilateral ovaries limitation patients, the only unilateral ovaries were took into statistics. While in bilateral ovaries limitation patients, we chose the compared lower dose side ovaries and took them into statistics. Below, the lower side ovarian maximum/mean dose means the maximum/mean dose of only unilateral ovaries or compared lower dose side in bilateral ovaries.

**Table 2:** Comparison of ovarian function in patients undergoing different radiation limitations

Limitation radiation to the ovary	No. of patients with normal ovarian function/no. treated	
No	0/13 (0%)	
Yes	41/105 (39.0%)	
Unilateral limitation	16/48 (33.3%)	
Bilateral limitation	25/57 (43.9%)	P = 0.318

**Fig. 1:**
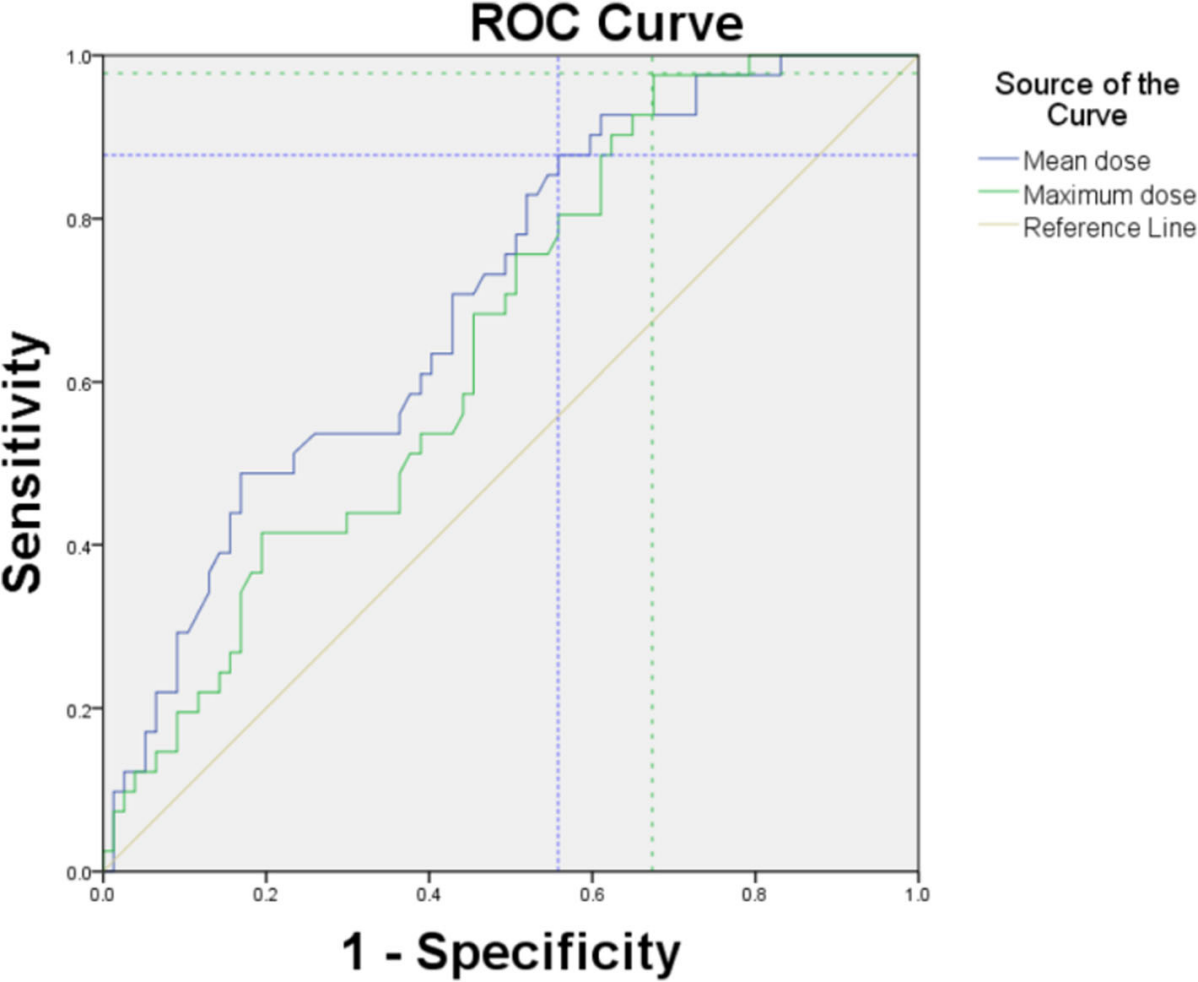
ROC curve statistics for determining the optimal ovarian limited dose. The blue color crossing dashed line denotes the cutoff value of mean dose and the green denotes the cutoff value of maximum dose

**Fig. 2:**
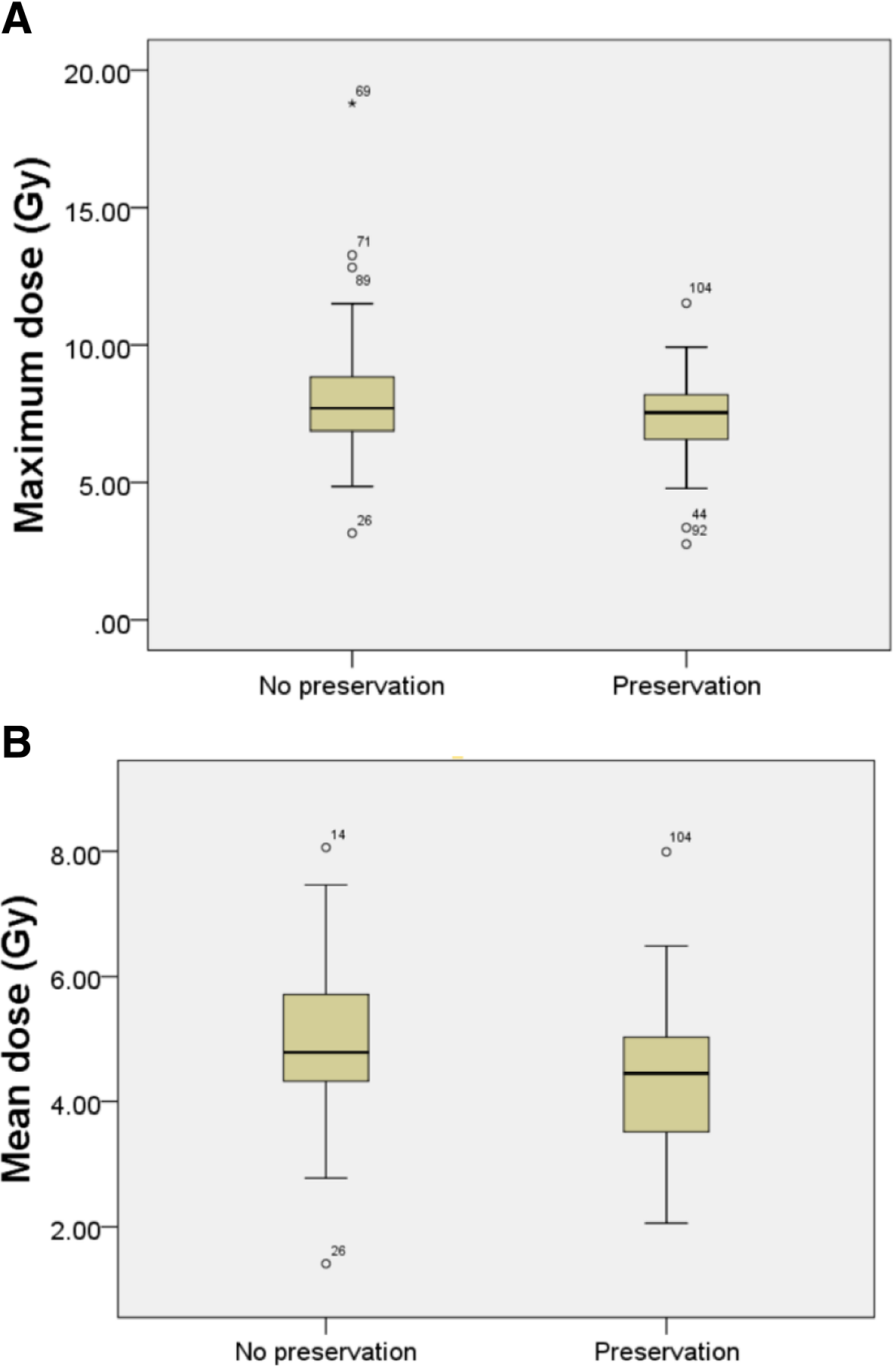
Maximum dose (**a**) and mean dose (**b**) distributions of patients with and without ovarian functional preservation

Using the area under the ROC curve and Youden index, we determined that the optimal limited radiation doses that are well tolerated by ovaries were Dmax< 9.985Gy and Dmean< 5.32Gy (Fig. 1), and the area under the curve was 0.654 and 0.704, respectively, while the 95% CI was 0.556–0.753 and 0.609–0.799, respectively. The lower side ovarian maximum dose of less than 9.985Gy was better at preventing the disruption of ovarian function. The lower side ovarian mean dose of less than 5.32Gy was better at preventing the disruption of ovarian function. Figure 2 indicated the dose distributions (max, mean) of patients with and without ovarian functional preservation.

**Fig. 3:**
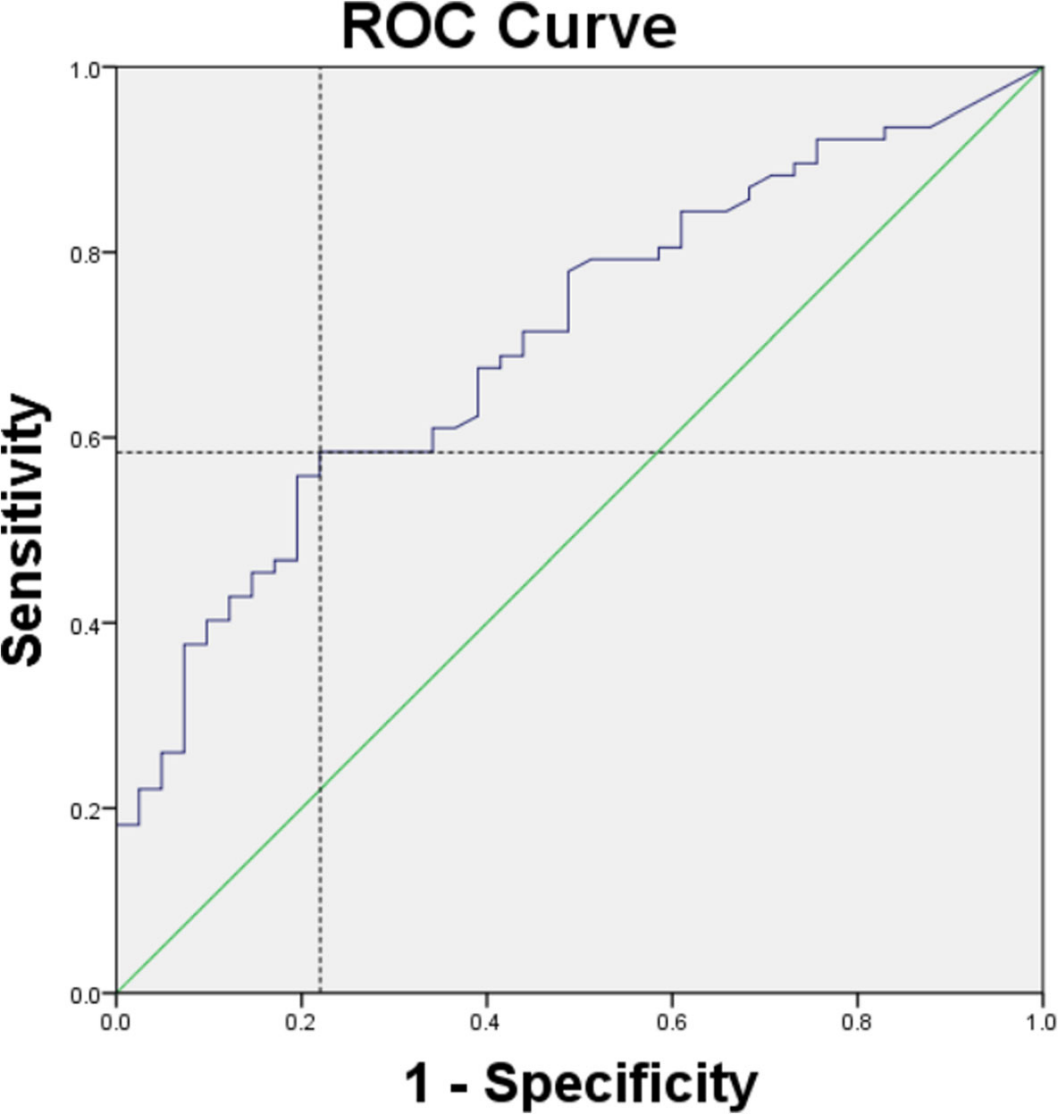
ROC curve statistics for determining the optimal dose–volume constrains. Area under the curve (AUC) is 0.706 (95% CI = 0.611–0.800). The blue crossing dashed line denotes the cutoff value of dose–volume

According to the cutoff of mean dose, by using area under the ROC curve and 95% CI statistical analysis, we determined that the optimal dose–volume constrains to ovaries was V5.5 < 29.65% (Fig. 3). The area under the curve was 0.706, and the 95% CI was 0.611–0.800.

Among ovarian dose limited patients, the average age of normal and abnormal ovarian function was 35.44 years and 39.09 years, respectively (*P* < 0.001). As shown in Table 3, patients were grouped by median age (38 years old), and we found that patients younger than 38 years old were more likely to keep normal ovarian function (*P* < 0.01).

**Table 3:** Comparison of ovarian function in patients of different age groups

Age	No. of patients with normal ovarian function/no. limitation treated	
≤38	30/57 (52.6%)	
>38	11/48 (22.9%)	P < 0.01

To investigate the correlation in unilateral/bilateral limitation, age and ovarian function, we used multivariate analysis and found that age ≤ 38 (*P* = 0.001, OR 0.240, 95%CI 0.100–0.578) was an independent predictors of ovarian function, while limited ovarian side numbers were excluded (*P* = 0.128, OR 0.514, 95%CI 0.218–1.211). (Table 4).

**Table 4:** Multivariate analysis of factor associated with ovarian function after lateral ovarian transposition

Charateristics	OR	95% CI	P value
Age (≤38/>38)	0.240	0.100–0.578	0.001
Unilateral/bilateral limitation	0.514	0.218–1.211	0.128

## Discussion

Recently, the incidence of cervical cancer in younger patients has increased, and the rationale behind ovarian transposition before radiotherapy is to maintain ovarian function for premenopausal patients. The incidence of cancer metastasis to the transposed ovaries could be thought of as rare and negligible [14, 15]. Morice P et al. found ovarian metastasis in just 1 out of 103 patients [16]. Only 3% of patients who had adnexal disease in transposed ovaries required analgesics or further surgery [17]. These results made maintain ovarian function for premenopausal patients by ovarian transposition possible and rationable.

Ovarian transposition before radiation therapy has been a hot issue for several years. In 2000, Buekers et al. reported that for patients who underwent ovarian transposition without radiation therapy, 98% of ovarian function was preserved for as long as 126 months after the procedure, and the average menopause age was 45.8 years. When radiation therapy was added, nearly 41% of ovarian function was preserved for an average period of 43 months, and the average menopause age was 36.6 years [18]. Hwang et al. reported that all eight patients who did not receive adjuvant radiation displayed normal ovarian function for more than one year when at least one of the ovaries was saved [11]. Feeney, D. D et al. found that without postoperative pelvic radiotherapy only 3/104(2.9%) patients who underwent lateral ovarian transposition experienced menopausal symptoms; however, only 14/28 (50%) of patients had preserved ovarian function when undergoing pelvic radiotherapy [17]. According to a review by Pahisa J, at a mean follow up of 44 months, 63.6% of patients who received radiotherapy and 93% of those patients without irradiation maintained normal ovarian function [19].

During the follow up period in the current study, we did not find ovarian metastasis in any of the 118 cases. Ovarian function was absent in 13 patients who received IMRT with no limitation on radiation dose to ovaries. Approximately 39.0% of patients who underwent IMRT with a limited radiation dose (as low as possible) to the ovaries had preserved normal ovarian function. According to these results, when postoperative radiotherapy is considered, ovarian transposition and dose limitation are needed to protect ovaries from radiation as ovaries are very sensitive to radiation-induced damage.

The international commission on radiation units and measurements (ICRU) recommends that planning organ at risk volume (PRV) margins should be used, because uncertainties and variations in the positioning of the organ at risk (OAR) during treatment must be considered to avoid serious complications [20]. A recent study proposed that the PRV margin for transposed ovaries is ~ 2 cm in all directions [21]; however, 2 cm margins would be excessively large in many cases. When the patient is younger, and the preservation of ovarian function is required, should the dose constraint of the ovarian PRV be considered as a priority, and what is the adequate dose constraint to transported ovaries?

In 2016, Zhenhua Du et al. reported on 21 patients who underwent IMRT with limited radiation dose (V10 < 20%) to the ovaries and sex hormone levels were measured. They found that limiting the ovarian radiation dose to V7.5 < 26% in IMRT can prevent the disruption of ovarian function, with the area under the curve being 0.740 and a 95% CI = 0.606–0.874 [22]. The samples used in this research were not enough, so we attempted to find the optimal radiation dose constraint to preserve ovarian function in IMRT.

It has been shown that without radiation treatment, ovarian endocrine function could be well preserved. In this study, ovarian function of 13 patients who received IMRT with no particular limitation on radiation dose to ovaries was absent, suggesting that ovarian function was significantly affected. Using IMRT, preservation of ovarian function should be possible when the limited dose was as low as possible to the ovaries.

There has been many discussions about ovarian preservation in the era of 2D opposite-field radiotherapy. In previous studies, the effectiveness of lateral ovarian transposition for ovarian preservation after adjuvant 2D radiation has been reported. The rate of ovarian failure varied widely from 17.0–88.6% after adjuvant radiation in patients after lateral ovarian transposition [15, 23, 24]. The widely varied result may be caused by many different influences such as the distance from iliac crest, uni- versus bilateral transposition, the age and so on.

Jong Ha Hwang et al. reported that 32.3% patients who received adjuvant radiotherapy had normal ovarian function regardless of the distance of translocated ovaries and the iliac crest [21]. Other research also reported that 63.6–71% of patients had preserved ovarian function after transposition of the ovary of more than 4~5 cm above the iliac crest [25]. In these cases in our study, translocated ovaries did not provide a satisfactory outcome, which may be aslo caused by the insufficient distance between ovaries and the radiation field.

In addition, bilateral ovary limitation may provide a better outcome compared to unilateral limitation. A total of 39.0% of patients in our study who underwent IMRT with a limited radiation dose (as low as possible) to the ovaries had preserved, normal ovarian function. The percentage of patients with normal ovarian function was 33.3 and 43.9% in unilateral and bilateral ovaries limitation (*P* = 0.318, Table 2), respectively. Bilateral ovary limitation seemed better than unilateral, but there was not statistical significance.

In addition, the relationship between age, limited ovarian side numbers and the success of ovarian transposition was observed. It had been reported previously that age was significantly correlated with ovarian function failure [16, 26]. Jong Ha Hwang et al. recommended bilateral ovarian transposition in patients who are< 32 years of age based on the ROC curve [11]. Clough KB. observed that the success rate was 100% for patients younger than age 40 years though laparoscopic unilateral ovarian transposition prior to irradiation. Only 2 patients older than 40 years were observed menopause among the 14 patients [14]. In our study, the average age of normal and abnormal ovarian endocrine function was 35.44 years and 39.09 years, respectively (*P* < 0.001). Multivariate analysis was used and age ≤ 38 years was one independent predictor of ovarian function (*P* = 0.001, OR 0.240, 95%CI 0.100–0.578) while limited ovarian side numbers were excluded, which means that transported ovaries in relatively younger patients may be more likely to be preserved in IMRT while unilateral/bilateral ovaries limitation did not appear to exert an obvious effect.

After all, the ROC curve method applied for further analysis indicated that the comparatively lower side ovarian maximum dose less than 9.985Gy, the mean dose of less than 5.32Gy, may be better at preserving ovarian function. These data suggest a new, optimal dose limit in IMRT to preserve ovarian function. The mean dose showed more area under the curve than the maximum dose. Limiting radiation dose to V5.5 < 29.65% in IMRT was the new option for the preservation of ovarian function. While the area under the ROC curve were not very good, only showed limited predictive value (around 0.70), which may be caused by sample-size restriction and the lack of stratification analysis that could affect the ovarian function.

## Conclusion

In summary, lateral ovarian transposition is an available method to preserve ovarian function in IMRT that still needs sufficient distance between ovaries and the radiation field. The selection of younger patients and adequate dose limitation of the transposed ovary during is required to maintain ovarian function effectively. Limiting the radiation dose to Dmax< 9.985Gy, Dmean< 5.32Gy and V5.5 < 29.65% in translocated ovaries in IMRT might be the new optimal option for the preservation of ovarian function, and there is no significant statistical difference between bilateral and unilateral limitations. However, there are still some limitations in our study, such as the lack of comparison between hormone levels before and after radiotherapy or at different follow up times. The results of our study may provide information for the design of future studies. Larger studies with a longer follow up time are needed to confirm the predictors for increased ovarian function preservation.

## Data Availability

The datasets used and/or analyzed during the current study are available upon reasonable request.

## Abbreviations

CI: Confidence interval
Dmax: Maximum dose
Dmean: Mean dose
E2: Estrogen
FIGO: Federation of Gynecology and Obstetrics
FSH: Follicle stimulating hormone
ICRU: International commission on radiation units and measurements
IMRT: Intensity-modulated radiotherapy
NCCN: National Comprehensive Cancer Network
OAR: Organ at risk
PRV: Planning organ at risk volume
PTV: Planning target volume
ROC: Receiver operator characteristic
V5.5: Percentage of volume with irradiated dose of 5.5 Gy
